# Management of Severe Traumatic Brain Injury: A Single Institution Experience in a Middle-Income Country

**DOI:** 10.3389/fsurg.2021.690723

**Published:** 2021-10-22

**Authors:** Ahmed Kamel Basha, Mohamed Ashraf Mahmoud, Mohamed Ismail Al Ashwal, Osama Aglan, Sherif Bahaa ElShawady, Assem Mounir Abdel-Latif, Ahmed M. Elsayed, Walid AbdelGhany

**Affiliations:** Neurosurgery Department, Ain Shams University, Cairo, Egypt

**Keywords:** traumatic brain injury, ICP monitoring, middle-income, management, decompressive surgery

## Abstract

**Introduction:** Severe traumatic brain injury (TBI) is a major public health problem usually resulting in mortality or severe disabling morbidities of the victims. Intracranial pressure (ICP) monitoring is recently recognized as an imperative modality in the management of severe TBI, whereas growing evidence, based on randomized controlled trials (RCTs), suggests that ICP monitoring does not affect the outcome when compared with clinical and radiological data-based management. Also, ICP monitoring carries a considerable risk of intracranial infection that cannot be overlooked. The aim of this study is to assess the different aspects of our current local institutional management of severe TBI using non-invasive ICP monitoring for a potential need to change our management strategy.

**Methods:** We retrospectively reviewed our data of TBI from June 2019 through January 2020. Patients with severe TBI were identified. Their demographics, Glasgow coma score (GCS) at presentation, treatments received, and imaging data were extracted from the charts. Glasgow outcome scale extended (GOS-E) at 6 months was also assessed for the patients.

**Results:** Twenty patients with severe TBI were identified on chart review. Ten patients received only medical treatment measures to lower the ICP, whereas the other 10 patients had additional surgical interventions. In one patient, a ventriculostomy tube was inserted to monitor ICP and to drain cerebrospinal fluid (CSF). This was complicated by ventriculostomy-associated infection (VAI) and the tube was removed. In our cohort, the total mortality rate was 40%. The average GOS-E for the survivor patients managed without ICP monitoring based on the clinical and radiological data was 6.2 at 6 months follow-up. The 6-month overall good outcome, based on GOS-E, was 33.3%.

**Conclusion:** Although recent guidelines advocate for the use of ICP monitoring in the management of severe TBI, they remain underutilized in our practice due to many factors. External ventricular drains were mainly used to drain CSF; however, the higher rates of VAIs in our institution compared with the literature-reported rates are not in favor of the use of ICP monitoring. We recommend doing a comparative study between our current practice using clinical-and radiological-based management and subdural or intraparenchymal bolts. More structured RCTs are needed to validate these findings in our setting.

## Introduction

According to the Centers for Disease Control and Prevention (CDC), traumatic brain injury (TBI) is defined as a bump, blow, or jolt to the head that disrupts the normal function of the brain (1). TBI is said to be severe when an extended period of unconsciousness or memory loss follows the injury (2). Severe TBI has been dubbed the term “silent epidemic” and is a major public health concern, usually resulting in mortality or severe disabling morbidities of the victims (3). Recent quantification of the global incidence of severe TBI in 2018 by Dewan et al. was estimated to be 5.48 million cases annually (73 cases per 100,000) (4). In addition, according to the WHO, it is expected that most severe TBI-related deaths occur in low and middle-income countries, where there is a relative paucity of evidence (5). This highlights the importance of contextualization of neurotrauma research as different regions have different needs and obstacles (6). Intracranial pressure (ICP) monitoring is recognized as an imperative modality in the management of severe TBI as dictated by Brain Trauma Foundation (BTF) Guidelines (7). On the contrary, Benchmark Evidence from South American Trials: Treatment of ICP (BEST TRIP) trial in the New England Journal of Medicine (NEJM) has questioned the external validity of the previously established BTF guidelines, suggesting a non-significant favorable outcome of ICP-based vs. clinically and radiologically-based management of severe TBI and no significant difference in hospital stay (8). In addition, there is some evidence in the literature that suggests that ICP monitoring does not affect the outcome when compared with clinical and radiological data-based management, as reviewed by Treggiari et al. (9). Harris et al.'s study is another similar study including 1,607 patients and concluding that no difference in outcome was observed in different healthcare settings of high-income and low-income countries (10). Also, ICP monitoring carries a considerable risk of intracranial infection that cannot be overlooked, with infection rate up to 7.29% in high-income countries as reported by Guyot et al. (11). Furthermore, it is important to mention that in the previously referenced study by Guyot et al. there was an association between the higher ventriculostomy-associated complication (12.04%) and GOS rather than the presenting Glasgow coma score (GCS) or the duration since the traumatic event. An independent-adjusted odds ratio of 4.3 for extracranial complications associated with ICP monitoring in pediatrics was reported by Salim et al. (high-income country) (12). Another complication is hemorrhage, which generally is of minimal clinical significance (13). Last, technical failure is a complication that is avoidable, early recognized, and poses minimal impact on the outcome (14). On the one hand, there is a strong body of evidence advocating the use of ICP monitoring as a basic standard of care, such as BTF guidelines and that in AL Saiegh et al. (36,929 patients) (15), on the other hand, ICP monitoring cost is high compared with non-invasive methods as shown in a recent study (16), where reported mean cost for ICP monitoring was 360,30 Mexican pesos vs. 356,37 for non-invasive methods. Also, the infection rate is higher, the application of EVD is difficult in normal-sized ventricles in absence of imaging assistance, and there is a lack of specialized neurosurgical critical care units (17).

Given the above, we conducted this study aiming to assess the different aspects of our current management of severe TBI for potential improvements and to question the external validity of using ICP monitoring as standard care in severe TBI cases in our setting.

## Materials and Methods

This is a single institution retrospective study that was carried out in Ain Shams University which is an academic public institution located in Cairo, Egypt. In this study, we retrospectively reviewed our data of TBI in Ain Shams University hospital from June 2019 through January 2020. All patients with severe TBI who visited our emergency department and were admitted to our neurosurgical service were included regardless their gender or age. Only patients with signs of brain stem death were excluded from our analysis. The demographics of the patients, GCS at presentation, treatments received including surgical interventions, ICP monitoring using external ventricular drain (EVD) when used, and imaging data were extracted from the charts. Glasgow outcome scale extended (GOS-E) at 6 months was also assessed. Descriptive statistical analysis was carried out using Microsoft Office Excel 2019. Our protocol for the management of severe TBIs is based on clinical data and interval imaging findings rather than ICP monitoring. Subdural or intraparenchymal bolts were not used for ICP monitoring as they are not nationally available for the management of severe TBIs. The only available method of ICP monitoring is ventriculostomy catheters that were used in cases with dilated ventricles mainly as a method of CSF drainage and also to monitor the ICP. This is primarily due to their difficult application in normal-sized ventricles in the absence of imaging assistance in our institution.

Our study protocol was reviewed and approved by the ethical board of the Neurosurgery department, and the study was approved by the Faculty of Medicine Ain Shams University Research Ethics Committee (FMASU REC). Written informed consent from the legal guardian/next of kin of the participants were not required to participate in this study in accordance with the national legislation and the institutional requirements since it is an observational retrospective study.

## Results

Among 156 patients with TBIs extracted from the charts of a 6-month period at AinShams University hospital, 20 patients with severe TBI were identified. Sixteen were men whereas four were women. The mean age was 29.5 years (± SD 22.8). The mode of trauma was as follows: fall from height in six patients, road traffic accidents (RTA) in 13 patients, and direct blow to the head with a blunt object in one patient (Table 1). The average duration of hospital stay was 16.65 days (± SD 12.8), whereas the average duration of intensive care unit (ICU) stay was 14.8 days (± SD 11.2). Median GCS at presentation to the emergency room (ER) was 7 (interquartile range 1.25) with none of the victims obeying commands on the best motor response. Nine patients were presented with GCS of 8, six patients with GCS of 7, two patients with GCS of 6, one patient with GCS of 5, one patient with GCS of 4, and one patient with a GCS of 3 with preserved corneal reflex and no signs of brain stem death at the time of presentation to the emergency department (ER). Nine patients (45%) had localizing brain injuries. In eight (40%) patients, one or both pupils were not reactive (Table 2). Median Rotterdam computed tomography (CT) score was four (interquartile range 2) at initial brain CT.

**Table 1:** Baseline characteristics.

**Baseline characteristics**	
**Age**	
Mean	29.5 years
SD	22.8 years
**Gender**	
Men no. (%)	16 (80%)
Women no. (%)	4 (20%)
**Mode of trauma**	
Fall from height no. (%)	6 (30%)
Road Traffic Accidents no. (%)	13 (65%)
Direct head trauma by blunt object no. (%)	1 (5%)

**Table 2:** Clinical characteristics.

**Clinical characteristics**	
**Duration of hospital stay (in days)**	
Mean	16.65
SD	12.8
**Duration of ICU stay (in days)**	
Mean	14.8
SD	11.2
**GCS at presentation to ER (median GCS 7)**	
GCS 8 no. (%)	9 (45)
GCS 7 no. (%)	6 (30)
GCS 6 no. (%)	2 (10)
GCS 5 no. (%)	1 (5)
GCS 4 no. (%)	1 (5)
Localizing brain injuries no. (%)	9 (45%)

Basal cisterns were compressed or absent in 75% of the patients (compressed in 35% while absent in 40%). A midline shift of more than 5 mm was present in half of the patients. The main pathology was brain contusion(s) in nine (45%) patients, acute subdural hematoma in six (30%) patients, epidural hematoma in two (10%) patients, and mixed pathology of acute subdural hematoma and brain contusion(s) in three (15%) patients (Table 3). All the patients in the group of brain contusion(s) had received medical treatments including measures to lower the ICP without any surgical interventions, except one patient who developed secondary posttraumatic hydrocephalus and was operated on for ventriculoperitoneal shunt.

**Table 3:** Imaging characteristics.

**Imaging characteristics**	
**Median Rotterdam score at initial brain CT: 4**	
**Basal cisterns**	
Compressed no. (%)	7 (35%)
Absent no. (%)	8 (40%)
Midline shift > 5 mm on initial brain CT no. (%)	10 (50%)
**Main pathology**	
Brain contusion(s) no. (%)	9 (45%)
Acute subdural hematoma no. (%)	6 (30%)
Epidural hematoma no. (%)	2 (10%)
Mixed pathology no. (%)	3 (15%)

All patients in the group of acute subdural hematoma had received surgical interventions in the form of decompressive craniotomy and hematoma evacuation, except one patient who was presented to ER with GCS 3 in which case the legally authorized representative refused the surgical intervention. In this group, five patients had supratentorial hematomas and only one patient had posterior fossa hematoma. This last patient was presented to ER with posterior fossa acute subdural hematoma and GCS of 8. He was operated on for posterior fossa decompressive craniectomy, left cerebellar acute subdural hematoma evacuation, and insertion of EVD through a Frazier burr hole into the dilated ventricles as a result of the presence of secondary obstructive hydrocephalus. This EVD was inserted mainly to drain CSF and was also used to monitor the ICP. One week after insertion of the EVD, ventriculostomy-associated infection (VAI) occurred as indicated by a CSF sample that was sent for laboratory workup. The culture showed no microbial growth. This can be justified by the fact that the patient was already on postoperative prophylactic antibiotics. This patient died 21 days after neurocritical care admission due to ventilator-associated pneumonia and systemic infection. In the group of mixed pathology of brain contusion(s) and acute subdural hematoma, two out of three patients had surgical interventions in the form of decompressive craniotomy and hematoma evacuation. The patient who was not surgically treated was unfortunately presented to the ER 8 h after he was hit by a car. His GCS at presentation was four with lateralizing signs, but his legally authorized representatives refused to do surgery. Two patients were presented with epidural hematomas and both of them were operated on for evacuation of traumatic space occupying hematomas.

In our cohort, the 30-day mortality rate was 40%. The average time to death was 9 days (±SD 5.26). The 6-month mortality was not changed from 30-day mortality (no mortalities added after the initial 30 days). Coexisting injuries were found in 11 cases (55%) and had aggravated the outcome in two (10%) of the patients. Chest infection was the most common complication occurring in four (20%) cases. Nervous system complications occurred in three (15%) patients. Two (10%) patients had developed early posttraumatic seizures and one patient developed posterior cerebral artery territory infarction as a result of uncal herniation.

Two patients (10%) were lost from follow-up. One of these two patients was presented with a GCS of 7 and his brain CT showed acute subdural hematoma, whereas the other patient was presented with an epidural hematoma and a GCS of 8. The average GOS-E for the survivor patients was 6.2 at 6 months follow-up. The 6-month overall good outcome based on GOS-E defined as patients with GOS-E of 7 or 8, was 33.3% (Table 4).

**Table 4:** Clinical outcomes.

**Clinical outcomes**	
30-day mortality no. (%)	8 (40%)
**Time to death**	
Mean	9 days
SD	5.26
**Complications**	
Chest infection no. (%)	4 (20%)
Early post-traumatic fits no. (%)	2 (10%)
PCA territory infarction no. (%)	1 (5%)
Lost follow up no. (%)	2 (10%)
**GOS-E for survivor patients at 6 months**	
Mean	6.2
Overall good outcome at 6 months no. (%)	6 (33.3%)

## Discussion

**Figure 1:**
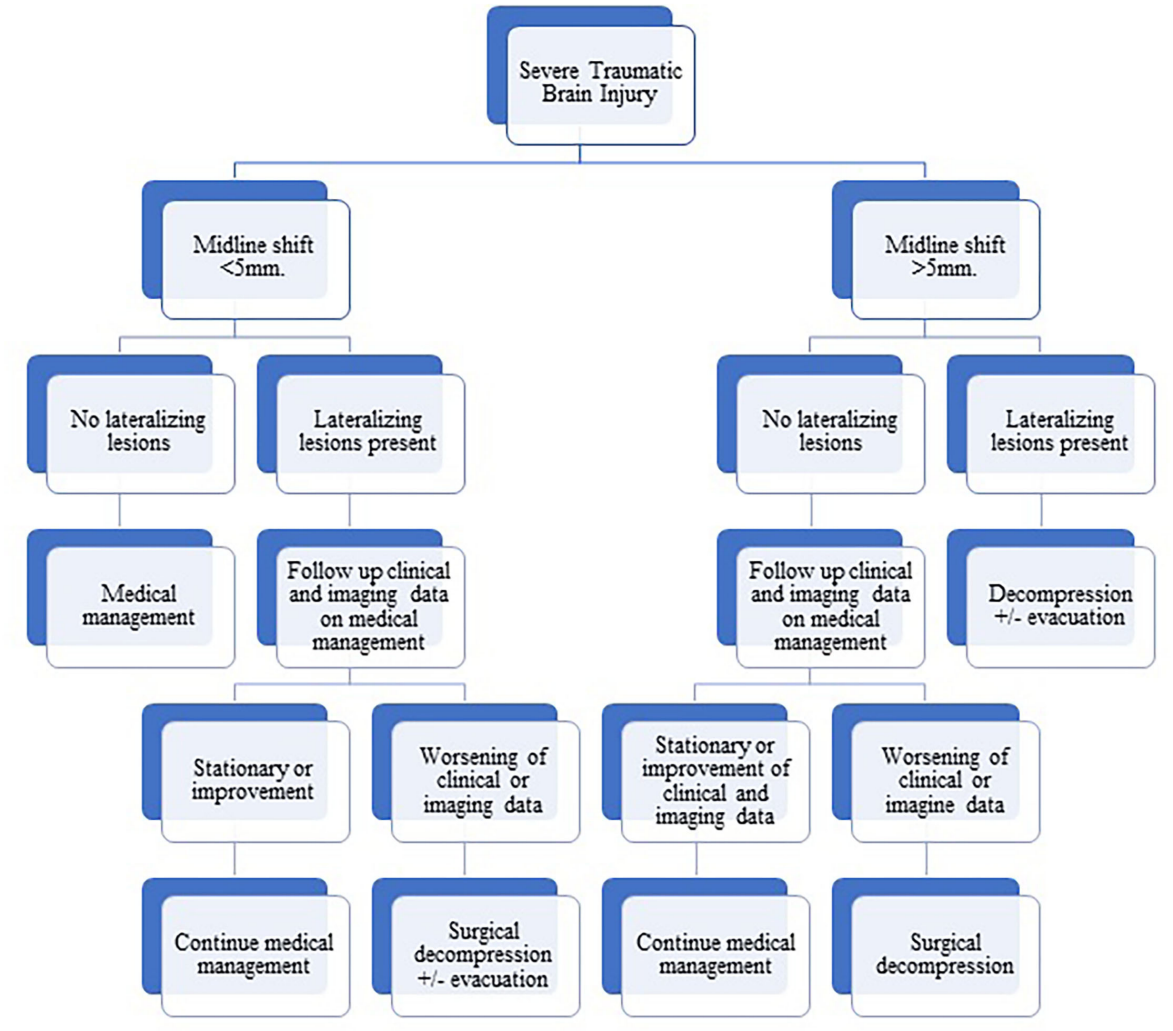
Flowchart diagram showing our protocol of management of severe traumatic brain injury based on clinical and radiological data.

In this work, we present our experience in the management of severe TBI as a part of the developing world where the ICP monitoring (standard of care)-based management is not available. Our protocol of management is based on clinical data and mainly brain imaging (see Figure 1). Surgical intervention in the form of decompression with or without evacuation of the mass lesion is offered for patients with lateralizing lesions and midline shifts more than 5 mm. In the absence of lateralizing brain lesions, an ascending sequence of medical (non-surgical) interventions is being adopted, including elevation of the head of the bed, hyperventilation, brain dehydrating measures including mannitol, and finally inducing barbiturate coma. On certain occasions, some patients without lateralizing lesions may shift from the non-surgical into the surgical group. For example, clinical deterioration despite full medical measures or occurrence of posttraumatic seizures with the development of malignant brain edema requiring decompressive surgery.

Our results are comparable to other clinical studies using either clinical and imaging-based management or using ICP-based management. In our experience, the 30-day mortality rate and 6-month mortality are the same since all mortalities occurred during hospital admission, which is comparable with Myburgh et al. (18). Furthermore, the GOS-E at 6 months is comparable to the findings as in Corral et al. (19). Despite these comparable results with reported literature, there is a paucity of comparative evidence between these two methods of management. The literature lacks well-structured randomized controlled trials to compare the two methods; however, this is due to ethical concerns of randomization (20).

**Figure 2:**
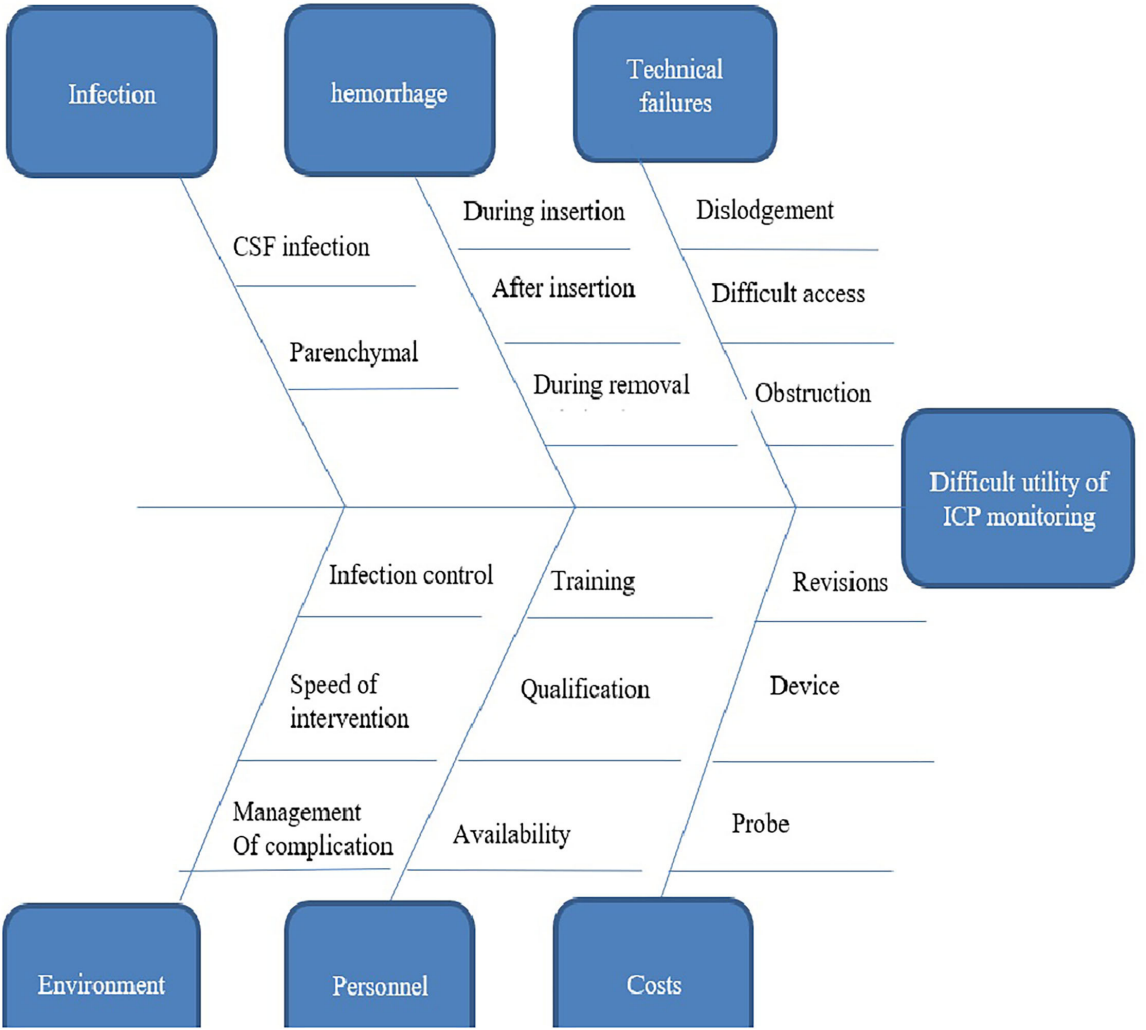
Fish bones diagram showing reasons behind the reluctance to use intracranial pressure monitoring based management of severe traumatic brain injury in our institute.

Given the unavailability of ICP-monitoring-based management, we seek to provide a fixed set number of patients with access to such care to compare with our otherwise conventional clinical-based protocol of management. This is a proposed solution to overcome the ethical notion of this comparison where all patients will receive the same standard of care in spite of having two comparison groups and then comparing the results of this set of patients with the results of conventional management. Reluctance to use ICP-monitoring-based management of severe TBIs could be explained by a variety of reasons (Figure 2). First, the a higher rate of infection from ventriculostomy catheters. The overall rate of VAI in our institution is 19.7%, which is higher in TBI cases reaching up to 27.9% (unpublished data) in comparison with 0–22% as reported by Sorinola et al. (21) with an average of 8.8% according to Tavakoli et al. (22) and 1–5% in Thailand (23). Second, the difficult EVD application in cases with normal ventricular size without imaging assistance, which also is not available in many institutions. Third, the evolution of growing evidence suggesting that there is no difference in outcome between ICP-monitoring-based management and clinical and imaging criteria-based management, which suppresses the motives behind shifting to adopt new management techniques. Fourthly, the unavailability of intraparenchymal and subdural bolts under insurance coverage in our country. Finally, the added costs in resource limited settings may have an impact on health care spending.

### Study Limitations

Limitations of our work include the small number of cases presented, the wide range of age distribution, and the lack of comparative methodology.

## Conclusion

Severe TBI is a major public health concern that causes significant mortality and morbidity worldwide (24). Although adopted as a standard of care in developed countries despite controversial evidence, the unavailability of ICP monitoring in developing countries poses the need for strong comparative evidence to advocate its use. Many factors raise concern for the use of ICP monitoring include infection rate, difficult application, high cost, etc. That said, we believe there is a strong need for a well-structured randomized body of evidence. We propose in the future providing a set of ICP monitoring in our institute to be compared with our conventional data.

## Data Availability Statement

The original contributions presented in the study are included in the article/supplementary material, further inquiries can be directed to the corresponding author/s.

## Ethics Statement

The studies involving human participants were reviewed and approved by Faculty of Medicine Ain Shams University Research Ethics Committee (FMASU REC). Written informed consent from the participants' legal guardian/next of kin was not required to participate in this study in accordance with the national legislation and the institutional requirements.

## Author Contributions

AB formulated the research question and participated largely in manuscript writing. MM participated in manuscript writing and editing. MA, OA, and SE participated in data collection. AE participated in the discussion and in the final revision of the manuscript. AA-L and WA participated in formulating the research question and in the final revision of the manuscript. All authors contributed to the article and approved the submitted version.

## Conflict of Interest

The authors declare that the research was conducted in the absence of any commercial or financial relationships that could be construed as a potential conflict of interest.

## Publisher's Note

All claims expressed in this article are solely those of the authors and do not necessarily represent those of their affiliated organizations, or those of the publisher, the editors and the reviewers. Any product that may be evaluated in this article, or claim that may be made by its manufacturer, is not guaranteed or endorsed by the publisher.

## Abbreviations

BTF: Brain Trauma Foundation
CDC: Centers for Disease Control and Prevention
CSF: Cerebrospinal fluid
CT: Computed tomography
ER: Emergency room
EVD: External ventricular drain
GCS: Glasgow Coma Score
ICU: Intensive care unit
ICP: Intracranial pressure
LMICs: Low- and middle-income countries
RCT: Randomized controlled trials
RTA: Road traffic accidents
TBI: Severe traumatic brain injury
SD: Standard Deviation.
